# Alveolar Macrophages in the Resolution of Inflammation, Tissue Repair, and Tolerance to Infection

**DOI:** 10.3389/fimmu.2018.01777

**Published:** 2018-07-31

**Authors:** Benoit Allard, Alice Panariti, James G. Martin

**Affiliations:** Department of Medicine, Meakins Christie Laboratories, Research Institute McGill University Health Centre, McGill University, Montreal, Quebec, Canada

**Keywords:** disease tolerance, macrophages, tissue damage control, pathogen persistence, lung

## Abstract

Pathogen persistence in the respiratory tract is an important preoccupation, and of particular relevance to infectious diseases such as tuberculosis. The equilibrium between elimination of pathogens and the magnitude of the host response is a sword of Damocles for susceptible patients. The alveolar macrophage is the first sentinel of the respiratory tree and constitutes the dominant immune cell in the steady state. This immune cell is a key player in the balance between defense against pathogens and tolerance toward innocuous stimuli. This review focuses on the role of alveolar macrophages in limiting lung tissue damage from potentially innocuous stimuli and from infections, processes that are relevant to appropriate tolerance of potential causes of lung disease. Notably, the different anti-inflammatory strategies employed by alveolar macrophages and lung tissue damage control are explored. These two properties, in addition to macrophage manipulation by pathogens, are discussed to explain how alveolar macrophages may drive pathogen persistence in the airways.

## Introduction

The lung serves the vital function of gas exchange, bringing oxygen to every single cell of the body, and disposing carbon dioxide. We inspire almost 11,000 l of air daily containing countless particles that include antigens, toxins, and microbes. It is remarkable that the lungs maintain a healthy and functional state, permitting in most instances considerable longevity. Ignoring harmless inhaled proteins, adapting to toxicants and limiting immune responses to bacteria and their cellular components are essential forms of adaptation that reduce tissue damage and that may be considered to be aspects of lung tolerance. However, while clearance of nocive inhaled substances is an optimal strategy, in some instances the host defense strategy decreases the host susceptibility to tissue damage but may permit pathogen survival. In other terms, disease tolerance is the result of the magnitude of the host reaction to the organism, which limits tissue damage but in doing so may fails to eliminate the pathogen. Here, we describe two important components leading to limiting of lung disease by alveolar macrophages (Aφs) by (i) repair of tissue damage and (ii) modulation of inflammation.

The Aφ is the first sentinel of the respiratory tree and constitutes the dominant immune cell in the steady state. These innate immune cells, derived from the yolk sac, are present as early as the first week after birth and are regulated in part by granulocyte-macrophage colony-stimulating factor (GM-CSF) (1, 2). Their niche in the alveolar space makes them important guardians of pulmonary homeostasis. Aφs regulate the response to infections and to epithelial damage. These functions require the engagement of different cellular pathways, one of which is pro-inflammatory and the other trophic, requiring a range of macrophage properties that often lead to a dichotomous classification of the Aφ phenotype.

The term macrophage (from Greek: μακρύς, makros = large and φαγειν, phagein = eater) was introduced by Elie Metchnikoff in 1883, following the description of the fundamental property of phagocytosis (3). A century later, macrophages had been observed in every single organ of the body and were recognized among the first actors of innate immunity. The ultra-structure of Aφs of mouse lung was described by Karrer (4) and the phagocytosis of India ink particles in the alveolar space by Aφs was observed 30 min after intranasal instillation. In the steady state, Karrer also observed a large amount of ferritin within Aφs suggesting that they ingested red blood cells. Sixty years later, the link between erythrocytes and macrophage biology has been established through the role of the heme signaling pathway in the development, differentiation, and function of macrophages (5). The most common function of Aφs phagocytosis is the removal of apoptotic cells to ensure tissue homeostasis. Extensive work by Fadok et al. described different receptors involved in this process (6). Aφs use different receptors such as immunoglobulin receptors and complement receptors to recognize opsonized microorganisms, facilitating their phagocytosis (7). The recognition of damage and pathogen-associated molecular patterns (DAMPs and PAMPs, respectively) by pattern recognition receptors, such as toll-like receptors or C-type lectin receptors, allows them to recognize the presence of pathogens or products of injury, and respond directly to provide optimal host protection (8). For instance, it has been recently shown that CD206 (mannose receptor) is involved in the recognition of *Mycobacterium tuberculosis* and the subsequent signaling (9). Aφs are also responsible for cleaning the epithelial environment by removing “waste materials” such as oxidized lipids using scavenger receptors. Notably, expression of MARCO and class A scavenger receptors (SR-AI/II) on Aφs is augmented so as to decrease pulmonary inflammation after oxidant inhalation (10). Finally, protection offered by pathogen recognition is complemented by enhancing the presentation of antigens to T cells. However, it seems that human Aφs are less efficient in this process due to a reduced expression of B7 costimulatory cell surface molecules (11), perhaps a useful characteristic in the avoidance of an exuberant response to harmless antigens.

## Macrophage Phenotypes

The opposing properties of Aφs designed to kill pathogens or to promote cellular proliferation and repair of tissues have been associated with supposedly discrete phenotypes termed the M1/kill and M2/repair macrophages (12, 13). Mills based this dichotomy on arginine metabolism: M1 can metabolize arginine to nitric oxide (and citrulline), an inhibitor of proliferation through cyclic guanosine monophosphate-dependent and -independent pathways (14), while M2 produce ornithine (and urea), a promoter of proliferation. Whether macrophages display an M1 or M2 profile is dependent upon the tissue environment as the tissue context may direct macrophages to provide an appropriate response (15). This plasticity results in a large spectrum of macrophage properties. In order to organize a classification of these macrophages, a consortium has published nomenclature and experimental guidelines (16).

Some of our understanding of the physiological functions of Aφs in the lung has resulted from observing the effects of their depletion. For example, the immunosuppressive properties of Aφs in the response to inhaled sensitizing proteins are manifested by prior depletion that results in an enhanced inflammatory response and an increased recruitment of antigen-presenting cells to regional lymph nodes and lung tissues (17). More recent studies confirm Aφ’s anti-inflammatory properties from the augmentation of inflammation in allergen challenged animals in which depletion has been induced prior to challenge (18, 19). Adoptive transfer of Aφs from allergen-resistant to allergen-susceptible rats prevents allergen-induced AHR and the inflammatory cytokines interleukin-13 and tumor necrosis factor-α (20). These findings indicate that quiescent Aφs have anti-inflammatory properties. Aφs harvested from allergen challenged animals are less effective in suppressing inflammation following adoptive transfer (18). The epithelial-derived alarmins IL-33 and TSLP promote the differentiation of quiescent Aφs to the M2 phenotype and augment macrophage-dependent allergic inflammation in the mouse (21, 22). Thus, Aφs show plasticity that is dependent on the microenvironment and whereas quiescent Aφs are predominantly immunosuppressive to avoid the development of unnecessary inflammatory responses to the host of inhaled foreign proteins encountered within the airway tree, when activated in the context of allergen challenge, the cells are less effective in their anti-inflammatory role.

## Alveolar Macrophages in Tissue Damage Control

**Figure 1:**
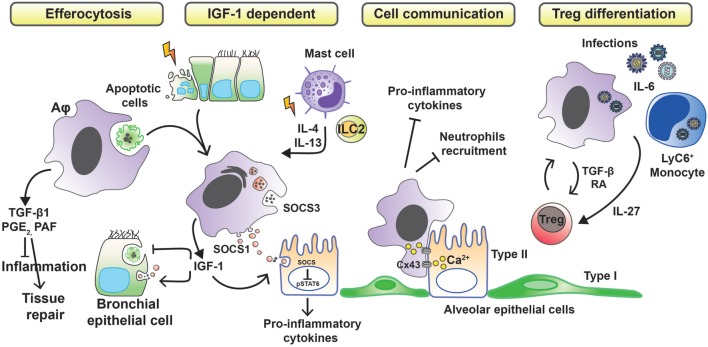
Anti-inflammatory strategies of alveolar macrophages favoring tissue damage control. Removal of apoptotic cells by Aφs (efferocytosis) leads to the secretion of anti-inflammatory mediators, such as transforming growth factor β1 (TGF-β1), prostaglandin E_2_ (PGE_2_), and platelet-activating factor (PAF), which in turn suppress the synthesis of pro-inflammatory cytokines, chemokines, and leukotriene C_4_. During phagocytosis of apoptotic cells or in response to inflammation-associated cytokines, Aφs also release insulin-like growth factor 1 (IGF-1). Binding of IGF-1 to its receptor on epithelial cells changes their phagocytosis pattern. Epithelial cells reduce the clearance of apoptotic cells while increasing the uptake of anti-inflammatory macrophage-derived microvesicles containing suppressor of cytokine signaling proteins (SOCS). Contact-dependent intercellular communication between Aφs and epithelial cells, using connexin 43 (Cx43)-containing gap junction channels, leads to synchronized calcium waves, using the epithelium as the conducting pathway and drives anti-inflammatory actions. Finally, Aφs promote the differentiation of regulatory T cells to further control inflammation.

Whether tissue damage is of infectious or inflammatory origin, Aφs must reduce the inflammation in first instance to limit the extent of injury. To do so, Aφs have been described to develop different anti-inflammatory strategies (Figure 1). Aφs are effectors of the resolution of inflammation through phagocytosis of apoptotic cells (efferocytosis), preventing dying cells from releasing pro-inflammatory and toxic contents into the environment while triggering the release of anti-inflammatory and repair factors (23). *In vivo* and *in vitro* studies have shown that apoptotic cell clearance induces the secretion of transforming growth factor β1 (TGF-β1), prostaglandin E_2_ (PGE_2_), and platelet-activating factor (PAF), with associated suppression of pro-inflammatory cytokines, chemokines, and leukotriene C_4_ (24–26). These findings have been confirmed in human. Indeed, defective lipopolysaccharide (LPS)-stimulated uptake of apoptotic cells by Aφs from patients with severe asthma has been associated with failure to induce the synthesis of PGE_2_ and 15-hydroxyeicosatetraenoic acid (15-HETE) (27). Moreover, defective phagocytosis has been observed in several respiratory pathologies. In severe asthma in children, macrophage function is abnormal and characterized by reduced phagocytic function and excessive apoptosis (28). In addition to asthma (27, 29), defective phagocytic function has been described in chronic obstructive pulmonary disease (30), cystic fibrosis (31, 32), and idiopathic pulmonary fibrosis (33) and also has been attributed a role in sustained/chronic inflammation.

Allergens, such as house dust mite, can cause apoptotic epithelial cell death (34) and trigger the synthesis of IL-4 and IL-13 from mast cells and type-2 innate lymphoid cells (ILC2s). These events lead to the production of insulin-like growth factor 1 from Aφs that enhances the uptake of anti-inflammatory macrophage-derived microvesicles by airway epithelium (35). Bourdonnay et al. report that Aφs can secrete suppressors of cytokine signaling SOCS1 and -3 in exosomes and microparticles, respectively, for uptake by alveolar epithelial cells and subsequent inhibition of STAT activation (36). Notably, airway epithelial cells can use PGE_2_ as a signal to evoke SOCS3 release from Aφs to dampen their endogenous inflammatory responses in an LPS inflammation model (37). Contact-dependent communication between Aφs and alveolar epithelium has been described also to modulate immunity through gap junction-like connections and the propagation of calcium waves (38). The consequence of this intercellular communication was immunosuppressive. The binding of CD200R and TGF-βR, expressed by Aφs, with their ligands (CD200 and TGF-β, respectively) present on the cell membrane of epithelial cells is a negative regulator of Aφ activation (15).

An alternative mechanism by which Aφs limit inflammation is through promoting a regulatory T cell (Treg) response. Cancer cell-activated M2-like macrophages induce activated Treg cells from CD4^+^CD25^−^ T cells *in vitro*. Interestingly, the authors also demonstrated a positive-feedback loop in which activated Tregs skewed the differentiation of monocytes toward an M2-like phenotype (39). Lung tissue-resident macrophages (Siglec F^+^ CD11c^+^ AutoFluorescent^hi^, likely Aφs) isolated from mouse and pulsed with ovalbumin when cocultured with antigen-specific CD4 T cells result in the generation of Foxp3^+^ Treg cells. Treg cell induction required both TGF-β and retinoic acid. Transfer of the antigen-pulsed tissue macrophages into the airways correspondingly prevented the development of asthmatic lung inflammation upon subsequent challenge with ovalbumin. However, other allergens, such as extracts from *Dermatophagoides pteronyssinus, Aspergillus fumigatus*, or cat dander, did not induce Tregs because of protease and TLR-mediated signals (40). Macrophages may also induce Tregs by an indirect pathway. Interleukin-6, a soluble mediator commonly associated with inflammation and elevated in humans with severe respiratory infection, is actually critical in promoting the resolution of the host response to respiratory viral infection and in limiting disease. Early, but not late, IL-6 signaling is required for the resolution of respiratory syncytial virus-induced immunopathology (41). Production of IL-6 after infection induces the production of the regulatory cytokine interleukin-27 by Aφs and recruited Ly6C^+^ monocytes, which in turn promotes the local maturation of Treg cells.

Since macrophages stand poised to rapidly produce large amounts of inflammatory cytokines in response to danger signals, it is logical that they are also the target of the process of resolution of inflammation. Indeed, several types of molecular controls work to downregulate the inflammatory responses of activated macrophages. These regulatory controls have been exhaustively reviewed by Mosser et al. (42). Regrettably, very few studies focused on Aφs are referenced, suggesting a gap in this field.

Once inflammation is controlled, tissue repair must take place to restore the normal tissue architecture. In the lung, the main cells damaged by infection and inflammation are epithelial cells. As long as the injury persists, pro-inflammatory signals continue, and further damage the epithelium. Thus, the repair process may be considered an integral part of the resolution of inflammation.

Important aspects of tissue repair by macrophages have been reviewed (23). Aφs with an M2 profile are the best candidates to orchestrate the repair of the epithelium since the metabolism of arginine to ornithine leads to cell proliferation and collagen production. Unexpectedly, M1 (or classically activated macrophages) may also participate in the lung repair by producing a large amount of amphiregulin in a mouse with LPS-induced acute lung injury (43). Amphiregulin, a ligand for the epidermal growth factor receptor, as well as other growth factors are necessary to ensure an optimal repair. Aφs produce these growth factors to counteract the epithelial damage induced by infection. For instance, Aφs that phagocytose apoptotic neutrophils produce hepatocyte growth factor (HGF) during bacterial pneumonia in mice (44). HGF is also produced by Aφs to enhance alveolar epithelial proliferation during influenza infection (45). Another major growth factor, also involved in tolerogenic response, is TGF-β1. Interestingly, macrophages that engage in efferocytosis may inhibit the TGF-β1 induced-epithelial–mesenchymal transition in lung alveolar epithelial cells *via* PGE_2_, PGD_2_, and HGF (46).

In studying the role of Aφs in lung physiology, precautions should be taken since the population of Aφs is heterogeneous. Indeed, monocyte-derived Aφs, recruited from the bone marrow during the inflammatory response, evoke different outcomes than resident Aφs. Monocyte-derived Aφs recruited in response to airway epithelial-derived monocyte chemoattractant protein 1/CCL2, are involved in airway inflammation and remodeling in allergic asthma (47). In a mouse model of lung injury (bleomycin and influenza A virus infection), monocyte-derived Aφs drive lung fibrosis and persist in the lung (48). However, monocyte-derived Aφs recruited after γ-herpesvirus (murid herpesvirus 4) infection may inhibit the development of house dust mite-induced experimental asthma (49). Thus, depending on the trigger for lung tissue damage and repair, Aφs but also monocyte-derived Aφs may have either beneficial or deleterious functions and more studies are required to better delineate the role of these macrophage subtypes in lung diseases.

## Tissue Damage Control may Drive Pathogen Persistence

The environment created by the tissue damage control may favor the persistence of pathogens in the airways. Indeed, the immunosuppressive properties of Aφs during the process of the control of tissue damage are presumably key in leading to immune evasion. Evasion from immune surveillance is an important parameter leading to the persistence of pathogens (50). The incidence of methicillin-resistant *Staphylococcus aureus* (MRSA) pneumonia in otherwise healthy individuals is increasing (51). These bacteria persist in lower airways by surviving within Aφs. An *in vitro* study found that *S. aureus* persists and replicates inside a murine Aφ cell line (52). Among the mediators used by Aφs to control tissue damage, we previously mentioned that PGE_2_ is produced after efferocytosis and exerts anti-inflammatory effects. PGE_2_ is known to suppress natural killer cell activity by increasing cellular cyclic adenosine monophosphate (53) and downregulates MHC class II expression on dendritic cells to decrease antigen presentation (54). More recently, it has been shown that the anti-inflammatory action of PGE_2_ in the lung is mediated only by the prostaglandin E receptor 4 (EP4) (55). In this way, it seems pathogens can take advantage of PGE_2_. Indeed, PGE_2_ can inhibit bacterial killing by Aφs by inhibiting NADPH oxidase (56). In macrophages infected by *M. tuberculosis*, PGE_2_ generated by TLR2 stimulation/p38 MAPK phosphorylation triggers EP4 to produce increased amounts of PGE_2_. Then PGE_2_ provides protection against necrosis *via* EP2 (57). Production of PGE_2_ by the host is a protective mechanism against *M. tuberculosis* by inhibiting type I IFN (58) as well as inducing apoptosis in macrophages (59, 60). Similarly, Influenza virus induces PGE_2_ to suppress type I IFN subverting innate immunity (61). Taken together, it seems that pathogens have developed mechanisms to induce PGE_2_ production by macrophages to suppress inflammation and better survive within the host. A recent study by Roquilly et al. shows that dendritic cells and macrophages developing in the lungs after the resolution of a severe infection acquire tolerogenic properties that contribute to persistent immunosuppression and susceptibility to secondary infections (62).

Aφ plasticity associated to the control of tissue damage is an important factor in pathogen persistence. The prevalence of the so-called M2 phenotype has been often associated with a positive outcome because of its ability to control tissue damage. However, M2 macrophages represent a permissive niche for the persistence of many intracellular pathogens (63). Indeed, persistence of bacteria has been described for several human diseases including Legionnaires’ disease (64) and tuberculosis (65). Alarmins, such as IL-33, IL-25, and TSLP, play an important role in macrophage polarization during tissue damage (66). The synthesis of IL-33 by epithelial cells, characteristic of the lung environment after birth, triggers the release of IL-13 by ILC2s and induces an anti-inflammatory M2 phenotype. Such an environment has been associated with the delayed response to *Streptococcus pneumoniae* infection in mice (67).

Decreased antimicrobial activity and augmented oxidative metabolism of M2 macrophages compared to glucose-dependent metabolism of M1 cells represent the main factors contributing to pathogen persistence in the host. The decreased production of nitric oxide following IL-4-driven arginase-1 expression facilitates the survival of pathogens sensitive to this reactive species (68) and perhaps explains why *Chlamydia pneumoniae* has been reported to prefer the M2 than M1 macrophage for its proliferation *in vitro* (69). In this scenario, pathogens not only benefit from but also drive macrophages toward the M2 phenotype that better suits their own requirements, as suggested by recent publications. A mathematical model has been proposed to facilitate the investigation of M1 to M2 switching following infection of macrophages with *M. tuberculosis* (70).

*Mycobacterium tuberculosis* upregulates the expression of peroxisome proliferator-activated receptor-γ in infected macrophages leading to increased lipid droplet formation, expression of M2 markers and downregulation of the M1 response, including the respiratory burst and nitric oxide production (71). In this way, *M. tuberculosis* not only circumvents the protective host response but may also guarantee the nutrient rich environment required for its growth and survival. Indeed, *M. tuberculosis* secretes a hydrolase to catalyze host lipid hydrolysis (72). This capacity of pathogens to use cell metabolism to persist in the airspaces seems unavoidable. Further, M2 macrophages demonstrate an iron metabolism of benefit for pathogens. M2 macrophages have reduced iron storage and increased iron and heme uptake resulting in a high iron label pool (73), thus favoring the growth and survival of pathogens (63). For instance, *M. tuberculosis* can use macrophages as an iron source and produce siderophores able to sequester iron from host transferrin and lactoferrin, leading to augmentation of iron concentrations in infected macrophages and favoring its growth (74). Other metal metabolism can be “highjacked” by pathogens, such as zinc. Vignesh et al. have shown that IL-4, a well known M2-polarizing signals, alters macrophage zinc homeostasis *via* metallothionein 3 and the zinc transporter SLC30A4, promoting pathogen persistence in M2 macrophages (75).

## Conclusion

Taken together, these studies demonstrate that Aφs have a central place in lung disease tolerance by (i) involvement in limiting lung tissue damage from potentially innocuous stimuli (ii) decreasing immune surveillance and (iii) by hosting pathogens. Pathogen persistence in the respiratory tract is an important preoccupation, and of particular relevance to conditions such as tuberculosis. Indeed, the equilibrium between the elimination of pathogens and maintenance of tissue integrity is a sword of Damocles for susceptible patients. Better understanding of the mechanisms of disease tolerance and in the appropriate setting breaking this tolerance may provide therapeutic options. An important field requiring further exploration is the discrimination of the role of resident macrophages versus recruited macrophages in the lung environment. How recruited macrophages interfere with various functions of resident Aφs to conserve the lung homeostasis is of great interest.

This study was funded by the Richard and Edith Strauss Canada Foundation.

## Author Contributions

BA, AP, and JM have written, discussed, and approved the final manuscript.

## Conflict of Interest Statement

The authors declare that the research was conducted in the absence of any commercial or financial relationships that could be construed as a potential conflict of interest. The handling Editor declared a shared affiliation, though no other collaboration, with the authors.
